# A Conversational Artificial Intelligence Agent for a Mental Health Care App: Evaluation Study of Its Participatory Design

**DOI:** 10.2196/30053

**Published:** 2021-12-01

**Authors:** Morena Danieli, Tommaso Ciulli, Seyed Mahed Mousavi, Giuseppe Riccardi

**Affiliations:** 1 Speech and Interactive Signal Lab Department of Engineering and Computer Science Università degli Studi di Trento Trento Italy; 2 IDEGO srl Rome Italy

**Keywords:** mental health care, conversational AI, mHealth, personal health care agents, participatory design, psychotherapy

## Abstract

**Background:**

Mobile apps for mental health are available on the market. Although they seem to be promising for improving the accessibility of mental health care, little is known about their acceptance, design methodology, evaluation, and integration into psychotherapy protocols. This makes it difficult for health care professionals to judge whether these apps may help them and their patients.

**Objective:**

Our aim is to describe and evaluate a protocol for the participatory design of mobile apps for mental health. In this study, participants and psychotherapists are engaged in the early phases of the design and development of the app empowered by conversational artificial intelligence (AI). The app supports interventions for stress management training based on cognitive behavioral theory.

**Methods:**

A total of 21 participants aged 33-61 years with mild to moderate levels of stress, anxiety, and depression (assessed by administering the Italian versions of the Symptom Checklist-90-Revised, Occupational Stress Indicator, and Perceived Stress Scale) were assigned randomly to 2 groups, A and B. Both groups received stress management training sessions along with cognitive behavioral treatment, but only participants assigned to group A received support through a mobile personal health care agent, designed for mental care and empowered by AI techniques. Psychopathological outcomes were assessed at baseline (T1), after 8 weeks of treatment (T2), and 3 months after treatment (T3). Focus groups with psychotherapists who administered the therapy were held after treatment to collect their impressions and suggestions.

**Results:**

Although the intergroup statistical analysis showed that group B participants could rely on better coping strategies, group A participants reported significant improvements in obsessivity and compulsivity and positive distress symptom assessment. The psychotherapists’ acceptance of the protocol was good. In particular, they were in favor of integrating an AI-based mental health app into their practice because they could appreciate the increased engagement of patients in pursuing their therapy goals.

**Conclusions:**

The integration into practice of an AI-based mobile app for mental health was shown to be acceptable to both mental health professionals and users. Although it was not possible in this experiment to show that the integration of AI-based conversational technologies into traditional remote psychotherapy significantly decreased the participants’ levels of stress and anxiety, the experimental results showed significant trends of reduction of symptoms in group A and their persistence over time. The mental health professionals involved in the experiment reported interest in, and acceptance of, the proposed technology as a promising tool to be included in a blended model of psychotherapy.

## Introduction

### Background

During the past 10 years, a multitude of mental health apps have been made available in the market [1,2]. Their functionalities range from (1) delivering questionnaires for mood self-monitoring [3,4] and (2) providing recommendations for emotion regulation [5] to (3) engaging users in rule-based interactions [6], sometimes with the support of web-based scripted dialogs [7]. As the requirement for mental health services is widespread [8] and with the current COVID-19 pandemic creating a spike in demand (as stated by the World Health Organization surveys on October 10, 2020 [9,10]), there is a greater awareness of these apps among mental health professionals [11,12]. However, there is little consensus on the usability and effectiveness of such systems [13]. Some independent research studies observed that often there is poor engagement from patients in continuing to use the apps after a few attempts [1]; others report concerns from the point of view of security, privacy, and ethical implications [14,15].

An increasing number of review papers have studied the use of chatbots in mental health. Chatbots are an evolution of internet-mediated psychological interventions. Although the latter were developed for supporting psychological care by prescriptive models, chatbots aim to engage users in short *conversations* about their mental distress. In the mental health domain, chatbots are often based on scripted or *Eliza-style* dialogs [6,16]. Bendig et al [17] have analyzed the results from 10 pilot studies published between 2009 and 2018. The goal of these pilots was to assess user acceptance and effectiveness of the therapeutic recommendations, but many of them mostly included nonclinical samples. The meta-analysis by Bendig et al [17] supports the view that state-of-the-art mental health chatbots are still experimental and that little evidence for transferring results to real psychotherapy contexts is available. In addition, Lim and Penn [18], who studied the potential of the application of digital technology in schizophrenia therapy, have stressed the need for reliable data, and the recent review by Gaffney et al [19] has highlighted the need for relying on unbiased data. However, Gaffney et al [19] have also stressed the importance of focusing current research in this field on the identification of the key mechanisms of action of the conversational agent interventions. This is very important, and in our view this aspect may be improved by meeting 2 requirements; that is, on the one hand by basing the interaction model of conversational agents on principled theoretical explanations of psychological change and on the other hand by involving mental health professionals in the design studies of blended interventions. This paper takes both recommendations into careful consideration.

### Objective

It should be noted that in the crowded landscape of mental health apps, there is a lack of principled protocols for developing personal agent–driven mental health interventions. Moreover, the involvement of mental health professionals in the design of the apps is almost missing, both in the phase of setting the requirements and in the evaluation of outcomes.

In this paper, we describe the protocol we are applying to develop Therapy Empowerment Opportunity or TEO, a mobile personal health care agent (m-PHA) for mental health whose goal is to support patients dealing with the perception of augmented levels of stress and anxiety related to problems in their workplace. In particular, the goal of our research is to test a protocol for investigating the opportunity offered by the integration of artificial intelligence (AI)–enabled conversational technology into a protocolized model of psychological treatment of work-related stress with the aim of increasing personal coping resources. Although different psychological approaches to the treatment of stress and anxiety offer important insights into the roots of burnout and work-related stress, for example, individual psychology [20] and different declinations of psychodynamic theory [21,22], we chose to integrate the m-PHA support into a protocol for the prevention and treatment of work-related stress based on cognitive behavioral theory (stress management training [SMT] and cognitive behavioral therapy [CBT]).

CBT is based on the cognitive theory concept that psychological distress is maintained by internal (cognitive) factors and activated by external factors. Emotional distress and maladaptive behavioral reactions are caused by maladaptive cognitions [23,24]. Changing cognitions and thoughts can help to reduce symptoms [25]. The effectiveness of these treatments has been proved in several studies: 4 meta-analyses showed how CBT performed better than the other interventions in the treatment of occupational stress [25].

SMT programs are widely used for therapeutic purposes, with proven effectiveness. These programs combine specific techniques such as relaxation with CBT. This approach considers stress to be the imbalance between strong demands (external or internal) and few individual coping resources. The goal of SMT interventions is to reduce the intensity of demands and increase coping resources [26]. The delivery of SMT interventions within the framework of cognitive behavioral principles has been shown to be effective for managing psychological distress related to work [27].

**Figure 1:**
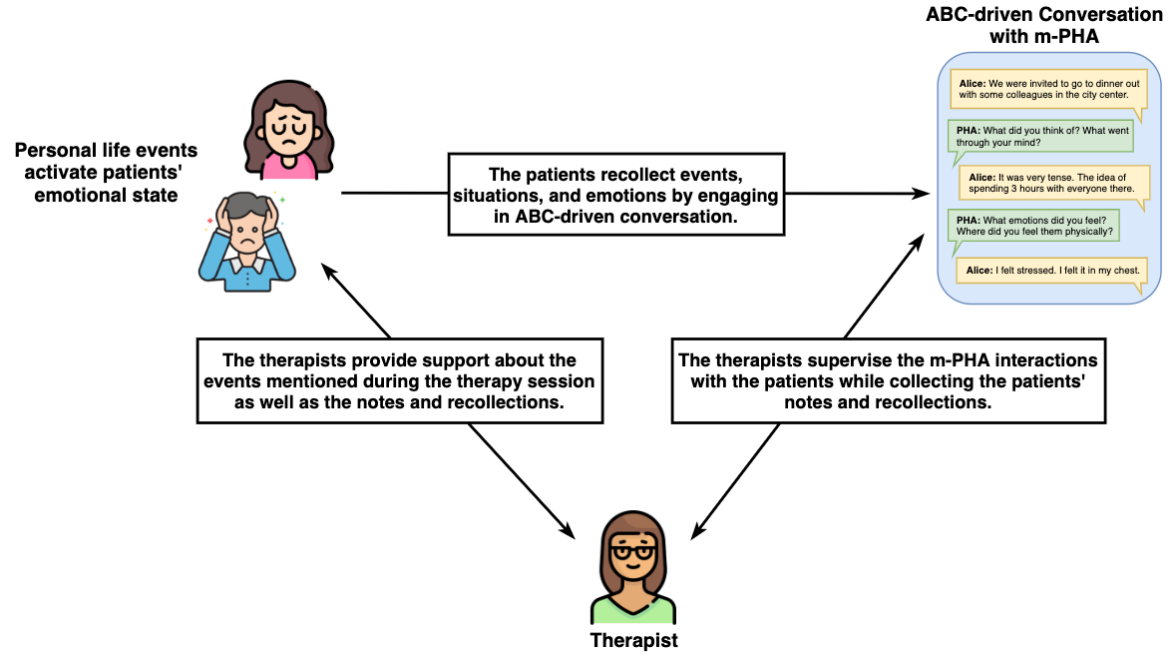
The information flow in the proposed approach. The patients interact with the mobile personal health care agent (m-PHA) to share personal recollections of their life events. The therapists supervise the interaction of the m-PHA with the patients and elaborate on the patients’ personal narratives during the therapy session. ABC: antecedents, beliefs, and consequences; m-PHA: mobile personal health care agent.

The approach is novel because it aims to (1) design the conversational features of the m-PHA to allow a natural and personal conversation and (2) allow the therapist to monitor patients’ progress and difficulties during the time between a session and the one that follows. For this purpose, the m-PHA engages the patients in short conversations that are not scripted but are based on the recognition of their emotional state and on the understanding of the personal content written during the period of the intervention. For example, if the user reports issues in their relationship with colleagues—“Today was a bad day because my boss asked me to complete my assignment before the conveyed deadline”—the m-PHA asks contextually appropriate questions such as “You wrote that you had a bad day with your boss due to his request to finish a task in advance of the agreed time. What emotions did you have, what mental images and thoughts?” Figure 1 represents the information flow in the system architecture.

A group of CBT therapists was involved in the process of designing this protocol as they provided information for identifying the variables that could be more suggestive of possible effectiveness of the approach. On the basis of these preliminary investigations, we set our research questions about the effectiveness (in terms of symptom reduction) of the joint use of psychotherapy and m-PHA, its possible persistence over time, and the acceptance of this integrated model by users and clinicians. The study is part of the European Union–funded Horizon 2020 research project COADAPT, whose aim is to develop methodologies to reduce work-related stress in aging workers.

## Methods

**Figure 2:**
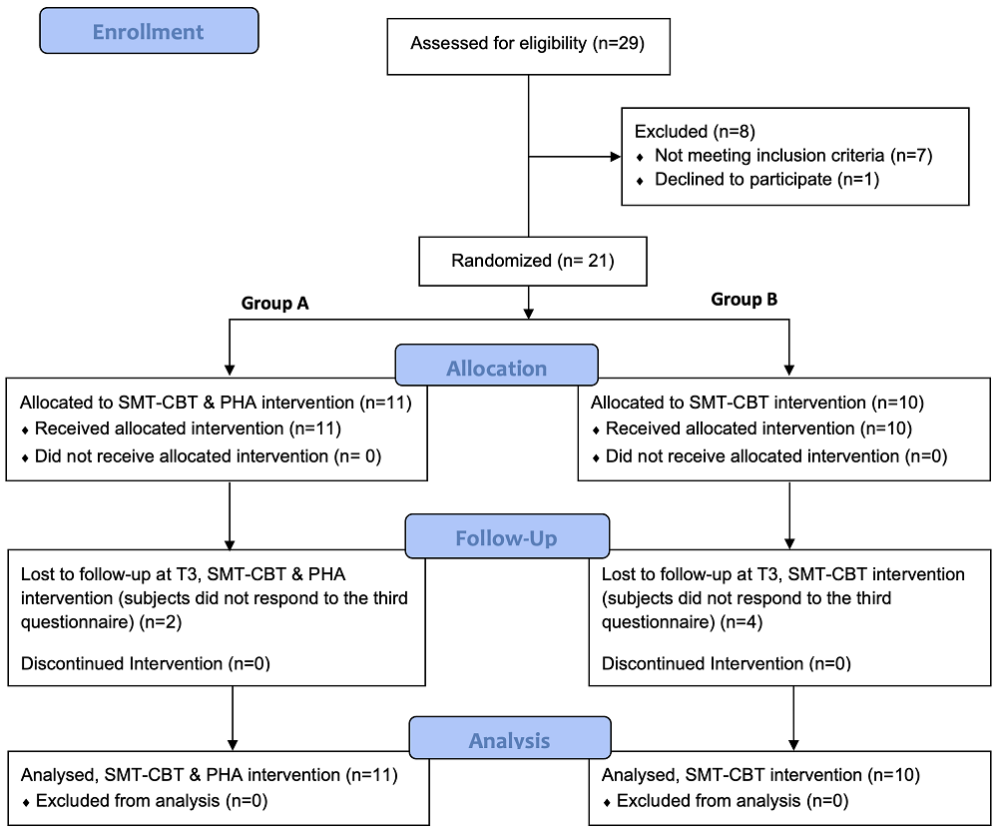
The CONSORT (Consolidated Standards of Reporting Trials) diagram shows the flow of the intervention, the enrollment of participants, their allocation to treatment, follow-up, and analysis. CBT: cognitive behavioral therapy; m-PHA: mobile personal health care agent; SMT: stress management training; T3: assessment of psychopathological outcomes 3 months after treatment.

The protocol and experimental plan were approved by the ethical committee of the University of Trento in Trento, Italy. The methodology of the intervention is described below and summarized in the CONSORT (Consolidated Standards of Reporting Trials) diagram (Figure 2).

### Recruitment

The study participants were recruited in Italy from aging workers who showed mild to high levels of distress or mild to moderate levels of anxiety and depression. The modalities for being enrolled in the study were described at psychoeducational seminars about work-related stress. A total of 160 workers participated in the seminars that were held at their workplace, of whom 64 (40%) showed interest in participating in the phases to follow of the protocol. Of these 64 workers, 29 (45%) decided to sign the informed consent forms and to undergo assessment of their levels of stress, anxiety, depression, and degree of well-being at their workplace. To select the participants, we administered the questionnaires described in the next paragraph. In addition, the participants tested negative for signs of mild cognitive impairment on the basis of the Montreal Cognitive Assessment (MoCA). The exclusion criteria included the presence of severe depression (Symptom Checklist-90-Revised [SCL-90-R] score >64), underlying psychiatric conditions, and neuropsychological mild impairment (MoCA score <26).

### Description of the Questionnaires for Initial and Final Assessment

The tests used for the initial assessment (T1) were the Italian versions of the SCL-90-R [28,29], the Perceived Stress Scale (PSS) [30,31], and the Occupational Stress Indicator (OSI) [32,33]. The SCL-90-R is a 90-item self-administered questionnaire that assesses a broad spectrum of psychological problems and psychopathological symptoms, measuring both internalizing symptoms (depression, somatization, and anxiety) and externalizing symptoms (aggression, hostility, and impulsivity). The questionnaire assesses 9 primary symptom dimensions: somatization, obsessiveness-compulsiveness, interpersonal hypersensitivity, depression, anxiety, hostility, phobic anxiety, paranoid ideation, and psychoticism. There are 3 global indexes: Global Severity Index (GSI), Positive Symptom Total (PST), and Positive Symptom Distress Index (PSDI). The PSS is a widely adopted questionnaire for the measurement of psychological stress. It is a self-reported questionnaire that was designed to assess “the degree to which individuals appraise situations in their lives as stressful” [30]. The OSI is a test for the wide-ranging detection of psychosocial stress in organizations. The different sections that make up the test detect the causes of perceived stress, their consequences, and individual coping resources. A further element detected by the instrument is the evaluation of some personal characteristics that, more than other characteristics, can promote stress. The Italian version of the MoCA was administered for assessing the absence of mild cognitive impairment [34].

### Protocol

In all, 8 psychotherapy sessions with CBT therapists were held through videoconference on a weekly basis. During the first session, the patients were invited to use the m-PHA to complete the assignments they received during the sessions, which included the writing of ABC (antecedents, beliefs, and consequences) notes.

The ABC technique is used in CBT to make individuals aware of their thoughts and to help them understand the link among events (antecedents), thoughts (beliefs), and emotions and behaviors (consequences). The technique increases understanding of nonfunctional behaviors and irrational or dysfunctional beliefs. The ABC technique was initially introduced by Ellis [24] and subsequently taken up by Beck [23]. The basic theory is that it is not events (A) that directly generate certain emotions but how these events are cognitively processed and evaluated and how irrational or dysfunctional beliefs (B) influence this processing [35-37].

In this protocol, the m-PHA conversed with the users to give names to the emotions they felt, to recognize their physical manifestations, and to localize them in some part of their bodies. In addition, it could provide suggestions for doing relaxation exercises. At the end of the psychotherapy treatment (T2), the participants received the same questionnaires submitted at T1, with the exclusion of the neuropsychological assessment. After 3 months, the study participants were contacted again for the third assessment (T3). At the end of the intervention, the psychotherapists involved in the experiment were engaged in a focus group to collect their opinions about the feasibility of integrating the m-PHA into the SMT-CBT protocol they apply with their patients.

### Participants

Sample characteristics are described in Table 1. A total of 29 potential participants were examined, and 21 (72%) were recruited and distributed into 2 experimental groups: group A received SMT-CBT treatment and the opportunity to use the m-PHA, whereas group B received only the SMT-CBT treatment. Of the 21 participants, 11 (52%) were assigned to group A and 10 (48%) to group B. On average, group A participants were aged 46.9 (SD 5.89) years and had 22.18 (SD 8.06) years of work experience, whereas group B participants were aged 48.7 (SD 10.21) years and had 25.30 (SD 11.59) years of work experience. Assigning participants to a control group was not planned in this experiment because the total number of participants we targeted was small and the goal of this study was to assess acceptability of the blended model of psychotherapy and the possibility of psychotherapists including an AI-enabled app in their work with patients. On the basis of the results of this study, we have planned and designed further experiments (currently running) in which a subset of participants has been assigned to a control group.

**Table 1:** Sample characteristics (N=21).

Characteristic		Values
Age (years), mean (SD)		47.76 (8.07)
**Gender, n (%)**		
	Male	4 (19)
	Female	17 (81)
**Groups, n (%)**		
	Group A	11 (52)
	Group B	10 (48)
**Formal education, n (%)**		
	High school	7 (33)
	Degree	10 (48)
	Master’s degree or PhD^a^	4 (19)
**Marital status, n (%)**		
	Single	3 (14)
	Cohabiting	4 (19)
	Married	13 (62)
	Separated	1 (5)

^a^PhD: Doctor of Philosophy.

### Statistical Analysis

Statistical analysis was performed using nonparametric statistics for ordinal data. In addition, by following the suggestions made by an anonymous reviewer and by Sullivan and Artino [38], a parametric independent 2-tailed *t* test analysis of data was performed.

The nonparametric statistical analysis applied the Mann–Whitney test to assess the differences between group A and group B for the results reported in the SCL-90-R, OSI, and PSS tests. Nonparametric within-group differences were assessed by applying the Friedman test. Wilcoxon tests were used to follow up the within-group findings.

## Results

### Parametric Data Analysis

#### Overview

Parametric data analysis (independent *t* test) was performed on the collected data by comparing the differences between groups A and B with respect to the results obtained in the SCL-90-R, PSS (Table 2), and OSI (Table 3) questionnaires at T1, T2, and T3. For the OSI test, we only considered the scales regarding coping strategies such as home-work relationship, social support, logic, task oriented, involvement, and time.

**Table 2:** Parametric analysis of differences between group A (n=11) and group B (n=10) at baseline (T1), after 8 weeks of treatment (T2), and 3 months after treatment (T3): Perceived Stress Scale (PSS) and Symptom Checklist-90-Revised tests.

Scale			Group A, mean (SD)		Group B, mean (SD)		*t* test (*df*)		*P* value
**PSS**									
	T1	22.09 (2.21)		20.40 (6.83)		0.75 (10.7)		.47	
	T2	16.55 (5.45)		14.80 (5.45)		0.73 (19)		.47	
	T3	18 (7.32)		10.29 (6.63)		2.22 (15)		.04	
**GSI^a^**									
	T1	60.36 (6.82)		55.70 (7.92)		1.45 (19)		.16	
	T2	55.82 (6.82)		49.00 (8.60)		2.02 (19)		.06	
	T3	53.89 (8.71)		47.00 (6.13)		1.67 (13)		.12	
**PST^b^**									
	T1	62.82 (6.81)		58.20 (9.66)		1.28 (19)		.22	
	T2	60.73 (10.38)		51.00 (12.13)		1.98 (19)		.06	
	T3	57.00 (8.02)		48.83 (7.76)		1.96 (13)		.07	
**PSDI^c^**									
	T1	53.82 (5.64)		51.00 (8.01)		0.94 (19)		.36	
	T2	48.91 (4.57)		47.80 (5.31)		0.51 (19)		.61	
	T3	49.00 (6.04)		45.17 (4.21)		1.34 (13)		.20	
**Somatization**									
	T1	52.64 (7.45)		50.40 (8.51)		0.64 (19)		.53	
	T2	49.64 (6.45)		47.50 (9.19)		0.62 (19)		.54	
	T3	48.78 (7.17)		46.17 (9.56)		0.61 (13)		.56	
**Obsessiveness-compulsiveness**									
	T1	62.55 (8.95)		51.30 (7.63)		3.08 (19)		.006	
	T2	56.55 (8.78)		48.20 (9.78)		2.06 (19)		.05	
	T3	55.11 (8.04)		48.17 (7.52)		1.68 (13)		.12	
**Depression**									
	T1	63.27 (8.86)		57.80 (8.59)		1.43 (19)		.17	
	T2	57.36 (9.29)		51.70 (8.41)		1.46 (19)		.16	
	T3	57.44 (11.90)		47.33 (6.83)		1.87 (13)		.08	
**Anxiety**									
	T1	57.55 (10.21)		56.50 (9.17)		0.25 (19)		.81	
	T2	59.27 (9.09)		49.60 (6.15)		2.82 (19)		.01	
	T3	53.00 (6.67)		48.33 (3.50)		1.56 (13)		.14	

^a^GSI: Global Severity Index.

^b^PST: Positive Symptom Total.

^c^PSDI: Positive Symptom Distress Index.

**Table 3:** Parametric analysis of differences between group A (n=11) and group B (n=10) at baseline (T1), after 8 weeks of treatment (T2), and 3 months after treatment (T3): Occupational Stress Indicator test.

Scale			Group A, mean (SD)		Group B, mean (SD)		*t* test (*df*)		*P* value
**Social support**									
	T1	5.91 (1.51)		5.90 (0.99)		0.02 (19)		.99	
	T2	6.36 (2.16)		5.50 (1.35)		1.08 (19)		.29	
	T3	5.90 (2.18)		7.33 (1.03)		–1.77 (13.6)		.10	
**Task oriented**									
	T1	5.45 (1.51)		4.90 (1.37)		0.88 (19)		.39	
	T2	5.82 (1.33)		6.10 (1.97)		–0.39 (19)		.70	
	T3	5.30 (1.25)		7.50 (1.05)		–3.60 (14)		.003	
**Logic**									
	T1	5.36 (1.96)		4.80 (1.99)		0.65 (19)		.52	
	T2	5.55 (0.93)		5.90 (1.52)		–0.65 (19)		.52	
	T3	5.50 (1.58)		5.67 (1.37)		–0.21 (14)		.83	
**Home-work relationship**									
	T1	5.45 (2.16)		6.10 (1.79)		–0.74 (19)		.47	
	T2	5.64 (1.96)		6.20 (1.32)		–0.76 (19)		.45	
	T3	6.10 (1.45)		6.83 (1.17)		–1.05 (14)		.31	
**Time**									
	T1	5.00 (1.34)		5.20 (1.99)		–0.27 (19)		.79	
	T2	5.09 (1.87)		5.80 (1.32)		–0.99 (19)		.33	
	T3	5.10 (2.18)		6.50 (2.07)		–1.26 (14)		.23	
**Involvement**									
	T1	5.73 (1.95)		5.90 (1.10)		–0.25 (19)		.81	
	T2	5.82 (1.08)		5.60 (1.84)		0.34 (19)		.74	
	T3	6.00 (1.25)		7.67 (1.37)		–2.5 (14)		.03	

#### SCL-90-R and PSS results

At T1, the SCL-90-R obsessivity and compulsivity levels in group A (mean 62.5, SE 2.70) were significantly different from those in group B (mean 51.3, SE 2.41; *t*_19_=3.08; *P*=.006; *r*=0.56).

At T2, the SCL-90-R anxiety levels in group A (mean 59.3, SE 2.74) were significantly different from those in group B (mean 49.6, SE 1.94; *t*_19_=2.82; *P*=.01; *r*=0.54).

At T3, PSS levels in group A (mean 18, SE 2.31) were significantly different from those in group B (mean 10.3, SE 2.50; *t*_15_=2.22; *P*=.04; *r*=0.49).

#### OSI Coping Strategies Results

For the OSI coping strategies, only the task-oriented and involvement scales at T3 were significantly different. The task-oriented levels in group A (mean 5.3, SE 1.25) were significantly different from those in group B (mean 7.5, SE 1.05; *t*_14_=–3.60; *P*=.003; *r*=0.69). The involvement levels in group A (mean 6, SE 0.39) were significantly different from those in group B (mean 7.67, SE 0.56; *t*_14_=–2.50; *P*=.02; *r*=0.56).

### Nonparametric Data Analysis

#### Overview

Nonparametric data analysis (Mann–Whitney test) was performed on the collected data by comparing the differences between the groups with respect to the results obtained in SCL-90-R, PSS (Table 4), and OSI (Table 5) questionnaires at T1, T2, and T3. For the OSI test, we only considered the scales regarding coping strategies such as home-work relationship, social support, logic, task oriented, involvement, and time.

**Table 4:** Nonparametric analysis of differences between group A (n=11) and group B (n=10) at baseline (T1), after 8 weeks of treatment (T2), and 3 months after treatment (T3): Perceived Stress Scale (PSS) and Symptom Checklist-90-Revised tests.

Scale			Group A							Group B									*U* value			Z value			*P* value
			Mean (SD)		Median		Mean rank		Mean (SD)					Median		Mean rank									
**PSS**																									
	T1	22.09 (2.21)		23.00		11.50		20.40 (6.83)					21.00		10.45		49.50			–0.39			.72		
	T2	16.55 (5.45)		17.00		11.73		14.80 (5.45)					15.00		10.20		47.00			–0.56			.59		
	T3	18 (7.32)		18.50		11.15		10.29 (6.63)					13.00		5.93		13.05			–2.10			.03		
**GSI^a^**																									
	T1	60.36 (6.82)		62.00		12.82		55.70 (7.92)				58.00			9.00		35.00			–1.41			.17		
	T2	55.82 (6.82)		58.00		13.59		49.00 (8.60)				49.00			8.15		26.50			–2.01			.04		
	T3	53.89 (8.71)		52.00		9.67		47.00 (6.13)				45.00			5.50		12.00			–1.77			.09		
**PST^b^**																									
	T1	62.82 (6.81)		63.00		12.45		58.20 (9.66)				59.50			9.40		39.00			–1.13			.28		
	T2	60.73 (10.38)		62.00		13.55		51.00 (12.13)				50.50			8.20		27.00			–1.98			.05		
	T3	57.00 (8.02)		58.00		9.67		48.83 (7.76)				44.00			5.50		12.00			–1.77			.08		
**Obsessiveness-compulsiveness**																									
	T1	62.55 (8.95)		63.00		13.95		51.30 (7.63)				53.00			7.75		22.50			–2.30			.02		
	T2	56.55 (8.78)		56.00		13.68		48.20 (9.78)				47.00			8.05		25.5			–2.08			.04		
	T3	55.11 (8.04)		54.00		9.50		48.17 (7.52)				46.00			5.75		13.50			–1.60			.13		
**Depression**																									
	T1	63.27 (8.86)		64.00		12.73		57.80 (8.59)				59.00			9.10		36.00			–1.34			.19		
	T2	57.36 (9.29)		57.00		12.68		51.70 (8.41)				48.50			9.15		36.50			–1.31			.20		
	T3	57.44 (11.90)		55.00		9.94		47.33 (6.83)				44.50			5.08		9.50			–2.07			.04		
**Anxiety**																									
	T1	57.55 (10.21)		54.00		10.82					56.50 (9.17)		56.00		11.20		53.00			–0.14			.90		
	T2	59.27 (9.09)		62.00		14.18					49.60 (6.15)		49.00		7.50		20.00			–2.48			.01		
	T3	53.00 (6.67)		53.00		9.39					48.33 (3.50)		47.00		5.92		14.50			–1.48			.15		

^a^GSI: Global Severity Index.

^b^PST: Positive Symptom Total.

**Table 5:** Nonparametric analysis of differences between group A (n=11) and group B (n=10) at baseline (T1), after 8 weeks of treatment (T2), and 3 months after treatment (T3): Occupational Stress Indicator test.

Scale		Group A				Group B				*U* value		Z value		*P* value
		Mean (SD)	Median	Mean rank	Mean (SD)		Median	Mean rank						
**Social support**														
	T1	5.91 (1.51)	6.00	11.05	5.90 (0.99)		6.00	10.95	54.5		–0.04		.99	
	T2	6.36 (2.16)	7.00	12.95	5.50 (1.35)		6.00	8.85	33.50		–1.56		.13	
	T3	5.90 (2.18)	6.5	7.60	7.33 (1.03)		7.00	10.00	21.00		–1.00		.32	
**Task oriented**														
	T1	5.45 (1.51)	5.00	12.05	4.90 (1.37)		5.00	9.85	43.5		–0.84		.44	
	T2	5.82 (1.33)	6.00	10.14	6.10 (1.97)		6.00	11.95	45.50		–0.68		.53	
	T3	5.30 (1.25)	6.00	5.95	7.50 (1.05)		7.00	12.75	4.50		–2.85		.004	
**Logic**														
	T1	5.36 (1.96)	6.00	11.91	4.80 (1.99)		5.00	10.00	45.00		–0.73		.49	
	T2	5.55 (0.93)	5.00	10.09	5.90 (1.52)		6.00	12.00	45.00		–0.73		.5	
	T3	5.50 (1.58)	5.5	8.30	5.67 (1.37)		6.00	8.83	28.00		–0.22		.85	
**Home-work relationship**														
	T1	5.45 (2.16)	5.00	10.05	6.10 (1.79)		6.00	12.05	44.5		–0.75		.46	
	T2	5.64 (1.96)	6.00	10.27	6.20 (1.32)		7.00	11.80	47.00		–0.58		.58	
	T3	6.10 (1.45)	6.00	7.60	6.83 (1.17)		7.00	10.00	21.00		–1.00		.35	
**Time**														
	T1	5.00 (1.34)	6.00	10.86	5.20 (1.99)		5.00	11.15	53.5		–0.11		.95	
	T2	5.09 (1.87)	6.00	9.50	5.80 (1.32)		6.00	12.65	38.50		–1.23		.22	
	T3	5.10 (2.18)	5.00	7.40	6.50 (2.07)		6.50	10.33	19.00		–1.22		.23	
**Involvement**														
	T1	5.73 (1.95)	6.00	10.23	5.90 (1.10)		6.00	11.85	46.5		–0.63		.56	
	T2	5.82 (1.08)	6.00	11.09	5.60 (1.84)		6.00	10.90	54.00		–0.07		.95	
	T3	6.00 (1.25)	6.00	6.55	7.67 (1.37)		7.50	11.75	10.50		–2.19		.03	

#### SCL-90-R and PSS results

At T1, the SCL-90-R obsessivity and compulsivity levels in group A (median 63) were significantly different from those in group B (median 53; *U*=22.5; Z=–2.30; *P*=.02; *r*=–0.50).

At T2, other significant differences between group A and group B were observed. With respect to the GSI levels, group A (median 58) differed from group B (median 49; *U*=26.5; Z=–2.01; *P*=.04; *r*=–0.44). The subscale measuring the PST of group A (median 62) differed from that of group B (median 51; *U*=27; Z=–1.98; *P*=.05; *r*=–0.43). With respect to anxiety, group A (median 62) differed from group B (median 49; *U*=20; Z=–2.48; *P*=.01; *r*=–0.54), and with respect to obsessivity and compulsivity, group A (median 56) differed from group B (median 47; *U*=25.5; Z=–2.08; *P*=.03; *r*=–0.45).

At T3, the depression level in group A (median 55) differed from that in group B (median 44.50; *U*=9.5; Z=–2.07; *P*=.04; *r*=–0.45). As for the PSS test, only at T3 did the levels reported by group A (median 18.5) differ significantly from those reported by group B (median 13; *U*=13.5; Z=–2.10; *P*=.03; *r*=–0.46).

In summary, data analysis at T1 did not show any significant difference for the PSS and SCL-90-R tests between groups A and B, with the exception of the subscale obsessiveness-compulsiveness of the SCL-90-R test (lower levels are better; see Table 2). At T2 and T3 for the SCL-90-R test, data analysis showed some differences between the 2 groups. Participants assigned to group A seemed to report lower improvements (lower levels are better) than those assigned to group B at T2 for the GSI, PST, obsessiveness-compulsiveness, and anxiety scales and at T3 for the depression scale. For the PSS test, group B showed significant improvements (lower levels are better) than group A at T3 (Table 4).

#### OSI Coping Strategies Results

For the subscales of the OSI test, the task-oriented level in group A (median 6) was significantly different from that in group B (median 7; *U*=4.5; Z=–2.85; *P*=.004; *r*=–0.62), and the involvement level in group A (median 6) was also significantly different from that in group B (median 7.5; *U*=10.5; Z=–2.19; *P*=.02; *r*=–0.48) at T3. The analysis of the results at T1 and T2 reported in the OSI scale did not show other significant differences between group A and group B (Table 5). Participants assigned to group A reported lower OSI results than participants in group B in any subscale (higher levels are better), but only the task-oriented and involvement subscales significantly differed between the 2 groups at T3 (Table 5).

### Group A Within-Group Analysis

#### Parametric Data Analysis

A parametric data analysis (1-way repeated measures analysis of variance) was performed for comparing the different results reported in the participants in group A at T1, T2, and T3.

The level of PSS (*F*_2,18_=3.25; *P*=.06) and some SCL-90-R subscales (GSI, *F*_2,16_=2.80; *P*=.09; PST, *F*_2,16_=1.58; *P*=.24; somatization, *F*_2,16_=1.44; *P*=.27; interpersonal hypersensitivity, *F*_2,16_=0.95; *P*=.41; depression, *F*_2,16_=2.34; *P*=.13; anxiety, *F*_2,16_=1.05; *P*=.37; hostility, *F*_2,16_=0.43; *P*=.65; phobic anxiety, *F*_2,16_=1.13; *P*=.35; paranoid ideation, *F*_2,16_=1.26; *P*=.31; and psychoticism, *F*_2,16_=1.47; *P*=.26) did not significantly change over the 3 measures at T1, T2, and T3.

For the PSDI and obsessiveness-compulsiveness subscales of the SCL-90-R test, the results show significant change over time (PSDI, *F*_2,16_=6.47; *P*=.03, with moderate effect size η^2^p=0.35 and obsessiveness-compulsiveness, *F*_2,16_=6.58; *P*=.008, with large effect size η^2^p=0.49).

The level of the examined OSI subscales of participants did not significantly change over the 3 measures at T1, T2, and T3 (social support, *F*_2,18_=0.44; *P*=.65; task oriented, *F*_2,18_=0.49; *P*=.62; logic, *F*_2,18_=0.09; *P*=.92; home-work relationship, *F*_2,18_=1.03; *P*=.37; time, *F*_2,18_=0.04; *P*=.96; and involvement, *F*_2,18_=0.22; *P*=.80).

#### Nonparametric Data Analysis

A nonparametric data analysis was performed using the Friedman test (Pereira et al [39]) for comparing the different results reported in the participants in group A at T1, T2, and T3. The level of PSS (PSS, *χ*^2^_2_=5.3; *P*=.07) and some SCL-90-R subscales (PST, *χ*^2^_2_=4.2; *P*=.15; PSDI, *χ*^2^_2_=4.2; *P*=.14; somatization, *χ*^2^_2_=3.5; *P*=.20; interpersonal hypersensitivity, *χ*^2^_2_=0.8; *P*=.71; depression, *χ*^2^_2_=5.4; *P*=.08; anxiety, *χ*^2^_2_=1.8; *P*=.45; hostility, *χ*^2^_2_=0.8; *P*=.71; phobic anxiety, *χ*^2^_2_=2.3; *P*=.33; paranoid ideation, *χ*^2^_2_=1.7; *P*=.47; and psychoticism, *χ*^2^_2_=3.0; *P*=.25) did not significantly change over the 3 measures at T1, T2, and T3.

The GSI and obsessiveness-compulsiveness subscales of participants significantly changed over the 3 measures at T1, T2, and T3 (*χ*^2^_2_=6.4; *P*=.04; w=0.35 and *χ*^2^_2_=6.4; *P*=.04; w=0.35, respectively). Wilcoxon tests were used to follow up this finding. A Bonferroni correction was applied; therefore, all effects have been reported at a 0.0167 level of significance. It seemed that the GSI did not significantly change from T1 to T2 (*t*=11.50; Z=–1.92; *P*=.06), from T1 to T3 (T=8; Z=–1.72; *P*=.09), or from T2 to T3 (*t*=13; Z=–0.169; *P*=.93). The obsessiveness-compulsiveness levels did not significantly change from T1 to T2 (*t*=11; Z=–1.96; *P*=.05) or from T2 to T3 (*t*=20; Z=–0.30; *P*=.79), but there was a significant change from T1 (median 63) to T3 (median 54; T=0.0; Z=–2.37; *P*=.02).

In summary, in group A, only the obsessiveness-compulsiveness levels showed a significant decrease at T1 in comparison with T3. The levels of the examined OSI subscales of participants did not significantly change over the 3 measures at T1, T2, and T3 (social support, *χ*^2^_2_=0.6; *P*=.77; task oriented, *χ*^2^_2_=1.3; *P*=.55; logic, *χ*^2^_2_=0.7; *P*=.76; home-work relationship, *χ*^2^_2_=0.9; *P*=.66; time, *χ*^2^_2_=0.8; *P*=.71; and involvement, *χ*^2^_2_=0.3; *P*=.90).

### Group B Within-Group Analysis

#### Parametric Data Analysis

A parametric data analysis (1-way repeated measures analysis of variance) was performed for comparing the different results reported in the participants in group A at T1, T2, and T3.

The level of PSS (*F*_2,12_=3.56; *P*=.06) and some SCL-90-R subscales (PSDI, *F*_2,10_=2.54; *P*=.17; obsessiveness-compulsiveness, *F*_1.1,5.48_=2.86; *P*=.15; interpersonal hypersensitivity, *F*_2,10_=3.85; *P*=.06; hostility, *F*_2,10_=3.71; *P*=.06; phobic anxiety, *F*_2,10_=0.147; *P*=.86; paranoid ideation, *F*_2,10_=3.20; *P*=.08; and psychoticism, *F*_2,10_=2.77; *P*=.11) did not significantly change over the 3 measures at T1, T2, and T3.

For the GSI scale, the Mauchly test indicated that the assumption of sphericity had been violated, *χ*^2^_2_=7.1; *P*=.03; therefore, multivariate tests have been reported (ε=0.55). The results showed significant change over time, V=0.85, *F*_2,4_=11.78; *P*=.02, with large effect size, η^2^p=0.63.

For the PST, depression, somatization, and anxiety subscales of the SCL-90-R test, the results showed significant change over time (PST, *F*_2,10_=8.87; *P*=.006; η^2^p=0.64; depression, *F*_2,10_=5.84; *P*=.02; η^2^p=0.54; somatization, *F*_2,10_=5.56; *P*=.02; η^2^p=0.53; and anxiety, *F*_2,10_=5.18; *P*=.03; η^2^p=0.51).

The levels of the examined OSI subscales of participants did not significantly change over the 3 measures at T1, T2, and T3 (social support, *F*_2,10_=3.89; *P*=.06; time, *F*_2,10_=2.10; *P*=.17; and home-work relationship, *F*_2,10_=3.57; *P*=.70).

For the task-oriented, logic, and involvement subscales of the OSI, the results showed significant change over time (task oriented, *F*_2,10_=7.80; *P*=.009; η^2^p=0.61; logic, *F*_2,10_=9.54; *P*=.005; η^2^p=0.66; and involvement, *F*_2,10_=5.56; *P*=.02; η^2^p=0.59).

#### Nonparametric Data Analysis

A nonparametric analysis of data was performed using the Friedman test (Pereira et al [39]), which allowed us to compare the different results reported in group B at T1, T2, and T3.

The levels of PSS (PSS, *χ*^2^_2_=4.5; *P*=.11) and some SCL-90-R subscales (PSDI, *χ*^2^_2_=1.7; *P*=.52; somatization, *χ*^2^_2_=5.0; *P*=.09; obsessiveness-compulsiveness, *χ*^2^_2_=5.3; *P*=.07; interpersonal hypersensitivity, *χ*^2^_2_=5.7; *P*=.06; anxiety, *χ*^2^_2_=4.7; *P*=.11; hostility, *χ*^2^_2_=3.9; *P*=.15; phobic anxiety, *χ*^2^_2_=1.3; *P*=.59; paranoid ideation, *χ*^2^_2_=4.3; *P*=.14; and psychoticism, *χ*^2^_2_=4.3; *P*=.12) did not significantly change over the 3 measures at T1, T2, and T3. The GSI, PST, and depression subscales of participants significantly changed over the 3 measures at T1, T2, and T3 (GSI, *χ*^2^_2_=9.5; *P*=.005; w=0.79; PST, *χ*^2^_2_=9.0; *P*=.008; w=0.75; and depression, *χ*^2^_2_=7.9; *P*=.01; w=0.66). Wilcoxon tests were used to follow up this finding. A Bonferroni correction was applied; therefore, all effects have been reported at a 0.0167 level of significance. It seemed that GSI did not significantly change from T1 to T2 (T=8; Z=–1.99; *P*=.04), from T2 to T3 (T=5.50; Z=–0.54; *P*=.69), and from T1 to T3 (T=0.0; Z=–2.23; *P*=.03).

PST did not significantly change from T1 to T2 (T=6; Z=–1.96; *P*=.05), from T2 to T3 (*t*=10.50; Z=0.0; *P*=.99), and from T1 to T3 (T=0.0; Z=–2.21; *P*=.03). Depression did not significantly change from T1 to T2 (*t*=10; Z=–1.79; *P*=.08), from T2 to T3 (T=9; Z=–0.31; *P*=.81), and from T1 to T3 (T=0.0; Z=–2.02; *P*=.06). The levels of the examined OSI subscales of participants did not significantly change over the 3 measures at T1, T2, and T3 (home-work relationship, *χ*^2^_2_=0.5; *P*=.90 and time, *χ*^2^_2_=3.4; *P*=.18). The social support, task-oriented, logic, and involvement subscales significantly changed over the 3 measures at T1, T2, and T3 (*χ*^2^_2_=7.1; *P*=.03; *χ*^2^_2_=8.5; *P*=.01; *χ*^2^_2_=8.0; *P*=.01; and *χ*^2^_2_=7.5; *P*=.01, respectively).

Wilcoxon tests were used to follow up this finding. A Bonferroni correction was applied; therefore, all effects have been reported at a 0.0167 level of significance. Social support did not significantly change from T1 to T2 (T=6.5; Z=–0.85; *P*=.53), from T2 to T3 (T=0.0; Z=–2.12; *P*=.06), or from T1 to T3 (T=0.0; Z=–1.86; *P*=.13). Task oriented did not significantly change from T1 to T2 (T=3; Z=–2.16; *P*=.05), from T2 to T3 (T=5; Z=–0.71; *P*=.75), or from T1 to T3 (T=0.0; Z=–2.32; *P*=.03). Logic did not significantly change from T1 to T2 (T=5; Z=–1.87; *P*=.07), from T2 to T3 (T=3; Z=–1.34; *P*=.37), or from T1 to T3 (T=0.0; Z=–2.12; *P*=.06). Involvement did not significantly change from T1 to T2 (T=7.5; Z=–0.65; *P*=.66), from T2 to T3 (T=2.5; Z=–1.72; *P*=.16), or from T1 to T3 (T=0.0; Z=–2.25; *P*=.03).

### Qualitative Evaluation of the Intervention

A focus group with some therapists was organized with the purpose of identifying the requirements for improving the acceptance of the m-PHA in SMT-CBT–oriented psychotherapy intervention. We chose the focus group technique because in the past this method has been found appropriate for evaluating attitudes of health care personnel, among others [19,40]. A total of 5 therapists who participated in the experiment were recruited in the group; a sixth therapist who participated in the design phase of the protocol but did not take part in the experiment played the role of facilitator. In all, 2 focus group meetings were conducted in July and September 2020. The therapists ranged in age from 29 to 39 years, the mean age being 35.05 (SD 2.40) years, and their professional experience ranged from 4 to 10 years, with a mean of 6.62 (SD 1.92) years.

The themes for the group discussion were the usefulness of including m-PHA support in the therapeutic process, their impressions about how that modification of the usual setting had an impact on the psychoeducational goals of the intervention, and the usability issues of the mobile app. Data analysis was conducted on the transcribed answers and on the notes taken during the group sessions. The data analysis was performed by following the method adopted by Berland et al [40]. The transcripts were reviewed by 2 authors (MD and TC) of this study, both with competence in conducting focus groups. From the analysis, the following relevant themes were identified.

All focus group participants reported the general impression that the m-PHA could improve patients’ engagement in their therapy goals. In the therapists’ view, the process followed for integrating this mental health mobile app into their practice was effective because the system helped their patients to complete the homework assigned by the therapists, allowing them to receive assistance while writing their ABC notes. The therapists observed that in their general practice they would usually spend more time focusing on teaching their patients how to complete their ABC notes so that they could be reviewed during the first part of the next session. In this trial, the spare time afforded to the therapists was effectively used to focus on events and related mental states that had already been shared through the app by the patients. In general, they recognized that most of the patients receiving the support of the m-PHA progressed faster in terms of the acquisition of the psychoeducational techniques of stress management.

The focus group participants carefully examined the different aspects related to the patient-therapist working alliance concerning the common goal of acquiring attitudes that may contribute to reducing the impact of stress in the patients’ everyday lives. In their view, the introduction of the m-PHA had no negative impact on the establishment of the working alliance.

As for usability issues related to the m-PHA app, the therapists expressed interest in extending the m-PHA support to their patients by including assistance in completing other types of CBT techniques, for example, disputing, in addition to the present support provided for ABC notes.

## Discussion

### Principal Findings

The analysis showed some significant differences between the 2 groups. The parametric analysis as well as the nonparametric analysis showed that in the examined subscales of the SCL-90-R, OSI, and PSS tests, group B seemed to show greater improvements than group A. The effect size in the parametric and nonparametric analyses was very large in scales that are significantly different.

In the SCL-90-R, for the subscales GSI, PST, anxiety, and depression, group B participants reported better changes on average than group A participants.

For the obsessivity and compulsivity scale, it is difficult to make an interpretation of what emerged because the 2 groups were different even at T1.

With respect to the PSS, group B showed better improvements than group A, especially at T3 where the effect size was very large.

As reported in the *Future Research* section, the conclusion of the intervention coincided with Italy entering lockdown because of COVID-19, and in the following months, different restrictions were imposed at different locations. This may have caused the increase in the level of anxiety observed in group A at T2 but not in group B, and the same circumstances applied to the level of stress at T3.

The dimensions evaluated by the OSI test, in particular the ones related to coping strategies, showed better improvements for participants assigned to group B than for group A participants. This difference was significantly different at T3 only for the task-oriented and involvement subscales, and the effect size was very large.

In addition, with regard to the mean levels of the SCL-90-R, PSS, and OSI tests, an improvement trend may be observed from T1 to T2 and from T2 to T3 in both group A and group B.

In group A, the mean of the obsessivity and compulsivity and PSDI subscales showed a significant decrease (Table 2) between assessment times, with a moderate effect for PSDI and a large effect for obsessiveness-compulsiveness. With nonparametric analysis, only the obsessiveness-compulsiveness values decreased, with a moderate effect (Table 4).

In group B, the mean of the GSI, PST, depression, somatization, and anxiety subscales showed a significant decrease (Table 2) between assessment times, with a large effect, as was the case for the task-oriented, logic, and involvement subscales, with a large effect. With nonparametric analysis, none of the SCL-90-R or OSI scales seemed to improve significantly over time, although the effect size is large. This could be an indication that sample size had an impact.

### Future Research

The goal of this study is to evaluate a protocol for an intervention for the treatment of work-related stress and anxiety based on the integration of a conversational AI-empowered mobile app into traditional psychotherapy. To validate the protocol, we needed to collect data from real users to feed the machine learning algorithms of the conversational m-PHA. More importantly, we needed to collect feedback from the psychotherapists who were involved in this participatory design effort. The limited number of participants that we could enroll did not allow the allocation of participants in more than 2 experimental groups. The research described in this paper was the initial and exploratory phase of a larger intervention protocol that is currently registered in ClinicalTrials.gov (NCT04809090). This larger protocol includes a control group, whose participants do not receive any type of treatment, as well as a fourth group, whose participants receive only the support of the m-PHA.

At the time of the data collection described in this paper, the version of the m-PHA used had limited dialog capabilities. The m-PHA was not yet able to engage participants in extended conversations: it aimed mainly to motivate users to leave personal narratives to complete the ABC homework required by the SMT-CBT protocol. The data collected in this experiment, as well as the input provided by the psychotherapists, allowed us to increase the dialog capabilities of the m-PHA.

Moreover, it is important to consider the temporal context of the data collection: the intervention phase began in December 2019 and ended in March 2020, coinciding with the first wave of the COVID-19 pandemic, and all of Italy was in lockdown for the first time. In the following months, different restrictions were imposed at different locations. During the last therapy sessions, many participants reported COVID-19–related episodes in their ABC diaries. It is likely that the participants reported anxiety levels that in some cases exceeded what they reported at the beginning of the experiment, and this was mainly because of the tragic situation that suddenly changed their daily life and, in some cases, their working conditions. In the revised protocol, the data analysis will also address the variables related to the regional variability of the COVID-19 pandemic in Italy, including the impact of regional lockdown measures.

### Conclusions

The results of our study shed light on the perspectives of applying AI technologies in the field of mental health care. The goal of the work described in this paper is 2-fold. The first objective is to evaluate the intervention protocol for integrating an m-PHA into the therapeutic process. The intervention addressed work-related stress management and engaged mental health professionals in the design and test phase. This blended approach included remote sessions of traditional SMT-CBT treatment as well as the integrated support of an m-PHA. The other objective of this study is to collect natural language and behavioral data to train the machine learning algorithms of the conversational agent and to design the experimental protocol in view of the ongoing randomized controlled trial.

The results support the hypothesis that SMT-CBT treatment may be integrated into AI-based mental health agents. The therapists engaged in the participatory design model adopted in this study are in favor of it, and in particular they deem that receiving the continuous support of conversational AI technology may improve patients’ adherence to their recommendations. Although the statistical analysis of data collected in this study does not yet show a clear advantage deriving from this integration, the group whose participants received the support of the m-PHA showed a significant positive trend of reduction of symptoms related with obsessivity and compulsivity and positive symptom distress.

## Abbreviations

AI: artificial intelligence
CBT: cognitive behavioral therapy
GSI: Global Severity Index
MoCA: Montreal Cognitive Assessment
m-PHA: mobile personal health care agent
OSI: Occupational Stress Indicator
PSDI: Positive Symptom Distress Index
PSS: Perceived Stress Scale
PST: Positive Symptom Total
SCL-90-R: Symptom Checklist-90-Revised
SMT: stress management training
